# A new therapeutic strategy for spotted fever group rickettsioses: a proposal for evaluating combination therapy with tetracycline and fluoroquinolones

**DOI:** 10.3389/fcimb.2026.1798936

**Published:** 2026-03-24

**Authors:** Kazuhiro Itoh, Hiroshi Tsutani, Yasuhiko Mitsuke, Masamichi Ikawa, Hiromichi Iwasaki

**Affiliations:** 1 Department of Internal Medicine, NHO Awara National Hospital, Awara, Japan; 2 Department of Community Health Science, Faculty of Medical Sciences, University of Fukui, Fukui, Japan

**Keywords:** fluoroquinolone, Japanese spotted fever, Rocky Mountain spotted fever, spotted fever group rickettsioses, tetracycline

## Introduction

Spotted fever group rickettsioses (SFGR) are significant tick-borne diseases worldwide, attributed to various *Rickettsia* species (Blanton, 2019; Zhang et al., 2023). Even though the clinical signs are similar, the death rates are very different depending on the pathogen and the patient’s risk factors. In tropical and subtropical areas, such as Africa, the Mediterranean, and Brazil, tick-bite fever, Mediterranean spotted fever (MSF), and the highly lethal Brazilian spotted fever are all very common (Angerami et al., 2006; Bechah et al., 2012).

The Centers for Disease Control and Prevention (CDC) says that tetracycline antibiotics (such as doxycycline) should be given right away as the main treatment (CDC, 2025). Even with this standard of care, the death rate for severe SFGR, like Rocky Mountain spotted fever (RMSF), is still too high (5–10%) (Jay and Armstrong, 2020), showing how badly novel strategies are needed.

Insights derived from Japanese spotted fever (JSF), induced by *Rickettsia japonica*, may be crucial. A retrospective analysis demonstrated that the combination of tetracycline and a fluoroquinolone considerably decreased the duration of fever in comparison to tetracycline alone (Itoh et al., 2023). Due to the common Toll-like receptor 4 (TLR4)-mediated immune responses in SFGR (Rumfield et al., 2020; Voss et al., 2021), this efficacy may be translatable. This study combines clinical and molecular knowledge to put out a therapeutic hypothesis: that using tetracyclines and fluoroquinolones together is an effective way to treat severe SFGR.

## Common pathophysiology of spotted fever group rickettsioses

The pathogenesis of SFGR is driven by the replication of rickettsiae within vascular endothelial cells (Clifton et al., 2005). When the endothelium is injured, it becomes more permeable. This leads to edema, hypotension, and the characteristic rash associated with the illness (Jay and Armstrong, 2020). In severe instances, disseminated intravascular coagulation (DIC) and multiple organ failure may occur.

The immunological response initiates upon the detection of rickettsial lipopolysaccharide (LPS) by the TLR4/MD2 complex (Maeshima and Fernandez, 2013; Guillotte et al., 2018). This turns on the NF-κB pathway, which causes the release of inflammatory cytokines such as IL-1β, IL-8, and MCP-1 (Clifton et al., 2005; Rumfield et al., 2020). Dysregulated synthesis of these mediators, essential for defense, might precipitate a “cytokine storm,” resulting in fatal consequences (Karki and Kanneganti, 2021). This TLR4-dependent mechanism is common in SFGR, which makes it important to target this pathway for treatment. **Table 1** shows a comparison of the most important features of the major SFGR.

**Table 1:** Key characteristics of major spotted fever group rickettsioses (SFGR) and implications for combination therapy.

Feature	Rocky Mountain spotted fever	Japanese spotted fever	Implications for SFGR
Causative organism	*R. rickettsii*	*R. japonica*	Multiple *Rickettsia* spp. worldwide
Geography	USA, Latin America; high fatality in Brazil/Mexico	Japan, East Asia	Distribution expanding in tropical/subtropical regions
Mortality	5–10%; ≥30% if delayed	~4.1%	Severity driven by endothelial injury & cytokine storm
Pathophysiology	Endothelial infection → vasculitis	Same	Shared TLR4/NF-κB/MAPK pathways
Standard therapy	Doxycycline	Doxycycline/minocycline	Early tetracycline is essential
Combination therapy evidence	Limited	In retrospective case series, combination with fluoroquinolone shortened fever by ~1.5 days	Hypothesis-generating for severe SFGR
Mechanistic rationale	—	Dual blockade (FQ = upstream; TC = downstream)	Additive inhibition of TLR4 signaling demonstrated in THP-1 *in vitro*
Precautions	FQ concerns in MSF (*R. conorii*)	—	Pathogen differentiation crucial in co-endemic scrub typhus regions

FQ, fluoroquinolone; MAPK, mitogen-activated protein kinase; MSF, Mediterranean spotted fever; NF-κB, nuclear factor kappa B; SFGR, spotted fever group rickettsioses; TC, tetracycline; TLR4, Toll-like receptor 4.

## Foundational evidence from Japanese spotted fever

JSF, a type of SFGR caused by *R. japonica* that can make you very sick, is an important clinical model for studying how well combination therapy might work (Itoh et al., 2023). In severe cases of JSF, disseminated intravascular coagulation and multiple organ failure may ensue, resulting in fatal outcomes, with a recorded death incidence of roughly 4.1% in 2019 (Itoh et al., 2023).

Several case reports have demonstrated the efficacy of combining tetracycline and fluoroquinolone antibiotics for severe JSF; however, our retrospective study employing a mixed-effects model yielded robust quantitative results. The study demonstrated that the combination therapy group exhibited a significantly greater reduction in body temperature compared to the tetracycline monotherapy group on both day 3 (difference: –0.569 °C; 95% CI: –1.117 to –0.020; p = 0.042) and day 4 (difference: –0.628 °C; 95% CI: –1.184 to –0.073; p = 0.027) of treatment (Itoh et al., 2023). The combination therapy shortened the duration to defervescence by 1.5 days, a key measure for quantitatively evaluating treatment effectiveness in infectious illnesses (Itoh et al., 2024a).

However, the safety profile of this combination requires careful interpretation. While two retrospective database studies in Japan did not show a mortality benefit for the combination, one study indicated a potential risk of convulsions and higher mortality (Kutsuna et al., 2022; Yasuda et al., 2024). A granular analysis of these results, however, uncovers significant subtleties. The correlation with negative outcomes in the study conducted by Yasuda et al. was predominantly influenced by cases related to ciprofloxacin. Importantly, levofloxacin, which made up the majority of combination therapy cases (87.0%), did not lead to higher death rates or serious problems compared to tetracycline monotherapy (Yasuda et al., 2024). This suggests that the reported risks may be drug-specific—potentially linked to ciprofloxacin-induced toxin upregulation or interactions—rather than a class effect of fluoroquinolones when used in combination. Importantly, given that levofloxacin represented the vast majority of the combination group in our efficacy analysis as well, the significant reduction in fever duration (1.5 days) is effectively a demonstration of the clinical benefit of the tetracycline-levofloxacin regimen.

Moreover, it is imperative to confront a significant issue frequently highlighted in previous literature about the application of fluoroquinolones for rickettsioses. Botelho-Nevers et al. conducted a well referenced retrospective analysis on Mediterranean spotted fever (MSF), revealing that fluoroquinolone treatment was independently linked to heightened illness severity (Botelho-Nevers et al., 2011). It is essential to highlight that this study assessed fluoroquinolone monotherapy in comparison to standard tetracycline therapy. The adverse results presumably stemmed from the suboptimal rickettsicidal efficacy of fluoroquinolones, resulting in treatment failure, rather than a toxic synergy intrinsic to the medication class. Although a subsequent *in vitro* study suggested that ciprofloxacin may upregulate a toxin-antitoxin module in *R. conorii* (Botelho-Nevers et al., 2012), this effect has not been shown to adversely affect outcomes when a potent rickettsicidal agent such as doxycycline is simultaneously administered, as indicated by the safety of the levofloxacin combination in JSF data. Therefore, our proposal endorses combination therapy (tetracycline + fluoroquinolone) to exploit the immunomodulatory advantage, solely as an adjuvant to tetracyclines, and evidence of monotherapy failure should not inhibit this strategy.

Nevertheless, given the nuances in safety data, a prospective randomized controlled trial is necessary to definitively evaluate the advantages and disadvantages of this combination therapy, ideally utilizing levofloxacin, which has demonstrated both safety and potential efficacy in retrospective analyses. The findings supporting expedited fever control in JSF offer a noteworthy clinical justification for advocating this immunomodulatory strategy for other severe SFGR.

## Mechanism of action: a dual immunomodulatory strategy

**Figure 1:**
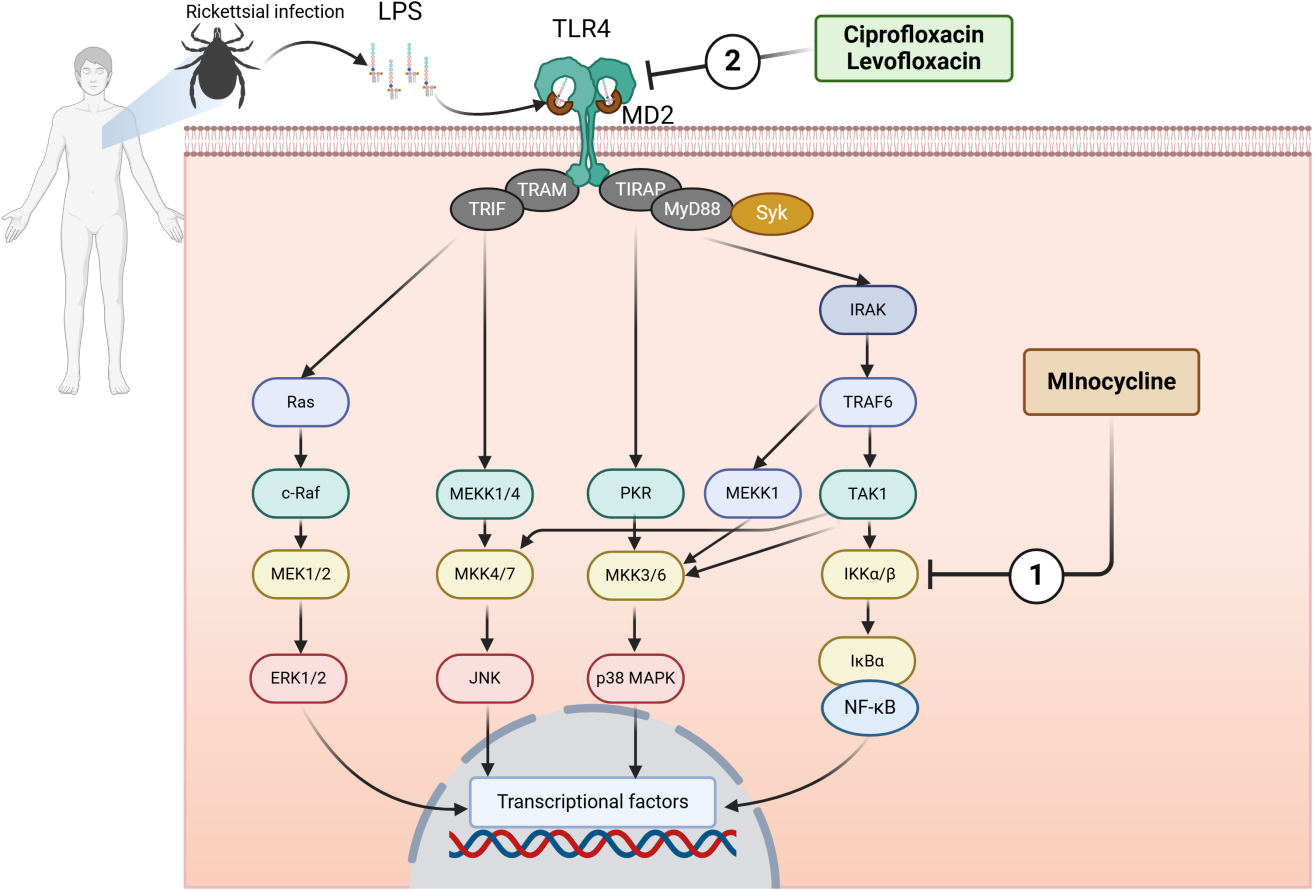
Dual immunomodulatory effects of tetracyclines and fluoroquinolones on TLR4 signaling in host immune cells. LPS from *Rickettsia* engages the TLR4/MD2 complex, leading to dimerization and activation of downstream signaling via NF-κB and MAPKs. 1) Minocycline inhibits phosphorylation of IKKα/β and prevents NF-κB activation, reducing TNF-α and chemokine production. 2) Ciprofloxacin and levofloxacin inhibit upstream LPS–TLR4/MD2 interaction and suppress phosphorylation of NF-κB p65 and ERK. Recent *in vitro* evidence shows that minocycline + ciprofloxacin produce additive inhibition of TNF-α, IL-8, IP-10, MIP-1α, and MIP-1β, and more strongly suppress NF-κB p65 activation than either drug alone in LPS-stimulated THP-1 cells. These findings support the concept of combined upstream and downstream blockade of the TLR4 pathway. ERK, extracellular signal-regulated kinase; IκBα, inhibitor of nuclear factor kappa-B kinase alpha; IKKα/β, IκB kinase alpha/beta; IL-8, interleukin-8; IP-10, interferon-inducible protein 10; IRAK, IL-1 receptor-related kinase; JNK, c-Jun N-terminal kinase; LPS, lipopolysaccharide; MAPK, mitogen-activated protein kinase; MEKK, mitogen-activated protein/ERK kinase kinase; MD2, myeloid differentiation factor 2; MIP-1α, macrophage inflammatory protein-1α; MKK, mitogen-activated protein kinase kinase; MyD88, myeloid differentiation factor 88; NF-κB, nuclear factor kappa-B; PKR, double-stranded RNA-activated protein kinase; Syk, spleen tyrosine kinase; TAK1, transforming growth factor-β-activated kinase 1; TIR, Toll/interleukin-1 receptor; TIRAP, TIR domain-containing adaptor protein; TLR, toll-like receptor; TNF-α, tumor necrosis factor-α; TRAF, tumor necrosis factor receptor associated factor; TRAM, TRIF-related adaptor molecule; TRIF, TIR domain-containing adaptor protein inducing interferon-β. The figure was created using BioRender (https://biorender.com/).

Although both tetracyclines and fluoroquinolones exhibit direct anti-rickettsial activity (Rolain et al., 1998; Satoh et al., 2019), the principal benefit of their concurrent application likely arises from their complementary immunomodulatory effects, which surpass their antibacterial capabilities. This approach fundamentally involves the simultaneous inhibition of the TLR4 signaling pathway, which is triggered by rickettsial LPS (Guillotte et al., 2018; Rumfield et al., 2020; Voss et al., 2021) (**Figure 1**).

These two groups of drugs regulate the host immune response through unique and complementary mechanisms.

1. Fluoroquinolones, including ciprofloxacin and levofloxacin, prevent the inflammatory cascade from taking place. They inhibit the first attachment of LPS to the TLR4/MD2 receptor complex on the surface of the host’s immune cells (Zusso et al., 2019). By obstructing this initial phase, they impede the subsequent signaling cascade, resulting in a reduction of inflammatory cytokines such as TNF-α and IL-1β in microglia and macrophage models (Zusso et al., 2019).

2. Tetracyclines, including minocycline and doxycycline, function inside cells and further down the signaling pathway. This is distinct from how other drugs work. They inhibit the phosphorylation of IκB kinase (IKK) α/β, which is the first step in starting the pro-inflammatory transcription factor NF-κB (Cazalis et al., 2008; Tai et al., 2013). By preventing NF-κB from being activated, they greatly reduce the production of many inflammatory cytokines and chemokines, including TNF-α, IL-1β, IL-6, IL-8, and MCP-1 (Cazalis et al., 2008; Tai et al., 2013).

The concurrent inhibition of both the upstream trigger (receptor binding) and the downstream signaling (intracellular transduction) by the combination of tetracycline and fluoroquinolone is hypothesized to produce a more extensive and potent suppression of the inflammatory response than either agent individually. Recent *in vitro* findings by Sakamaki et al. substantiate this hypothesis, demonstrating that the combination of minocycline and ciprofloxacin significantly enhances the inhibitory effect on TNF-α production and reduces inflammatory chemokines (IL-8, IP-10) in LPS-stimulated THP-1 monocytic cells, surpassing the effects of either drug alone (Sakamaki et al., 2025). This dual blocking is the proposed mechanism to avert the life-threatening cytokine storm and mitigate the significant vascular endothelial damage linked to advanced SFGR (Karki and Kanneganti, 2021).

## Discussion

Our opinion is that tetracycline–fluoroquinolone (TC+FQ) combination therapy merits formal evaluation as an adjunctive strategy for severe or high-risk SFGR. This hypothesis-driven approach is grounded in dual immunomodulatory blockade of the TLR4 pathway and supportive retrospective observations in JSF. However, translating this concept into feasible trials and responsible clinical use requires explicit operational plans for safety, target population, and a key diagnostic confounder: scrub typhus.

### Operationalizing the scrub typhus confounder in trials and practice

Because *Orientia tsutsugamushi* is intrinsically fluoroquinolone-resistant and rapid point-of-care diagnostics distinguishing SFGR from scrub typhus are not widely available in many endemic settings, enrollment and treatment algorithms must be designed for real-world constraints rather than assuming immediate microbiologic confirmation. Importantly, we do not assume that all SFGR share identical clinical features with JSF, nor do we propose extrapolating JSF-specific findings as universal rules for SFGR. Instead, we propose a pragmatic “enrichment + safety-switch” framework that can be adapted to local epidemiology and locally prevalent SFGR species.

First, clinical–epidemiologic enrichment should be region- and species-informed. Rather than enrolling all undifferentiated febrile rash illnesses, trial eligibility can be restricted to patients whose presentation is most consistent with SFGR in the given setting and less consistent with scrub typhus, using pre-specified criteria informed by local surveillance data and published clinical patterns. In regions where JSF is the predominant SFGR, enrichment criteria may incorporate JSF-based differentiating features as illustrative, locally relevant examples (e.g., palm/sole involvement reported more frequently in JSF cohorts, relatively small or inconspicuous eschars, and region-specific seasonality), while explicitly acknowledging that other SFGR species may present differently. In other geographic areas, enrichment criteria should be recalibrated to the dominant local SFGR species and their known clinical spectrum.

Second, mandatory confirmatory testing with a prespecified “rescue switch” is essential for safety. At enrollment, all participants should submit specimens for definitive testing (e.g., PCR from eschar/whole blood and/or paired serology). Because results may return after empiric therapy has started, the protocol should include a clear safety rule: if scrub typhus is confirmed—or becomes strongly suspected on follow-up based on evolving clinical features or available testing—the fluoroquinolone component must be discontinued immediately and patients should continue standard effective therapy, with predefined monitoring for clinical deterioration.

Third, analytic handling should be prespecified to preserve interpretability. Participants later confirmed as scrub typhus should be excluded from the per-protocol analysis and handled using a modified intention-to-treat (mITT) framework, complemented by sensitivity analyses using different case-definition thresholds. This ensures that pragmatic enrollment does not dilute efficacy estimates for SFGR while maintaining patient safety and transparency.

In short, we do not advocate empiric TC+FQ for all febrile rash illnesses. Rather, we propose a locally calibrated enrichment strategy coupled with mandatory confirmatory testing and an immediate fluoroquinolone discontinuation (“rescue switch”) rule to operationalize the confounder in settings where point-of-care differentiation is limited. We emphasize that enrichment criteria are intended to increase pre-test probability, not to replace microbiologic diagnosis, and must be updated as local epidemiology and diagnostic availability evolve.

### Who should be studied first

Because most uncomplicated SFGR respond to tetracycline alone, we propose evaluating TC+FQ primarily in severe/high-risk SFGR (e.g., early organ dysfunction, DIC tendency, encephalopathy, hypotension). In this subgroup, accelerated control of hyperinflammation may plausibly translate into clinically meaningful stabilization. The regimen should remain anchored on an effective tetracycline, and the fluoroquinolone component should be selected with caution given drug-specific concerns reported for ciprofloxacin in other rickettsioses.

### Safety oversight and feasibility

Future trials should incorporate structured safety monitoring (e.g., neurologic events, arrhythmia/QT risk where relevant), predefined stopping rules, and independent oversight. This framework allows the field to test the hypothesis responsibly while acknowledging diagnostic and operational realities in endemic regions.

## Conclusion and future directions

Even though the *Rickettsia* species that cause SFGR (such as RMSF and JSF) are different, they all have very comparable TLR4-dependent host immune responses and clinical presentations (Blanton, 2019; Jay and Armstrong, 2020; Itoh et al., 2023). The CDC (CDC, 2025) says that tetracycline antibiotics are the best treatment, however the high death rate in severe SFGR cases demonstrates how desperately we need new options (Jay and Armstrong, 2020). This study integrates modern clinical and molecular findings to offer an innovative therapeutic approach: the combination of tetracycline and fluoroquinolone antibiotics. The justification for the proposal is twofold. Recent clinical findings from JSF suggest that this combination therapy can induce defervescence more effectively than tetracycline monotherapy (Itoh et al., 2023, Itoh et al, 2024b). We propose that this accelerated defervescence is not solely a symptomatic improvement (i.e., a more rapid alleviation of sickness behavior) but rather a clinical marker of earlier regulation of the underlying immunological hyperactivity. The efficacy of employing early clinical response, including defervescence, as a therapeutic endpoint is well-documented in other acute bacterial infections, such as community-acquired pneumonia, where it is applied in clinical trials sanctioned by regulatory authorities like the U.S. Food and Drug Administration (FDA) and in associated analyses (Laessig, 2010; U.S. Food and Drug Administration, 2020). Although our 1.5-day reduction necessitates prospective validation to establish a direct correlation with decreased mortality, we hypothesize that the timely management of hyperinflammation is the essential initial measure in averting the advancement to severe consequences, including DIC and organ failure. The significant immunomodulatory effect of this combination—attained by simultaneously inhibiting the TLR4 inflammatory pathway at both upstream and downstream sites—offers a robust molecular basis for its potential effectiveness (Cazalis et al., 2008; Tai et al., 2013; Zusso et al., 2019). This dual blockage may be key in preventing the lethal cytokine storm and mitigating the significant vascular endothelial damage that contributes to mortality in these conditions (Karki and Kanneganti, 2021).

Prior to conducting large-scale trials, preclinical investigations utilizing proven animal models of severe SFGR would be essential to further evaluate the *in vivo* efficacy and mechanisms of this combination therapy. Models like the virulent *Rickettsia parkeri* infection model in C3H/HeN mice or the endothelial-targeting *R. conorii* model might be appropriate for this objective (Bechah et al., 2012; Londoño et al., 2019). Due to their shared endemic areas, SFGR and scrub typhus (caused by *O. tsutsugamushi*) are difficult to diagnose in clinical practice (Sakamaki et al., 2025). This is important since *O. tsutsugamushi* has a gyrA (Ser83Leu) mutation that makes it fluoroquinolone-resistant (Jang et al., 2013). Clinical worsening has been observed in scrub typhus patients receiving ciprofloxacin treatment (Jang et al., 2013). Accordingly, operationalization should rely on a tiered approach (clinical pre-screening/enrichment, mandatory confirmatory testing with a prespecified rescue switch, and mITT/per-protocol analytic handling), as detailed in the Discussion section. Given the limitations of retrospective investigations, a prospective, carefully designed, and rigorously controlled randomized controlled trial (RCT) is needed to confirm this therapeutic approach (Kutsuna et al., 2022; Yasuda et al., 2024). Regulatory authorities (FDA, 2020) have stated that rigorous studies are required to determine the efficacy and safety of novel antibacterial treatments for severe infections. The trial should be structured to assess the efficacy and safety of combination therapy, specifically in SFGR patients classified as high risk for severe disease. Based on epidemiological risk factors for mortality, we define “high-risk” patients as those of advanced age, those with delayed initiation of treatment, or those presenting with early signs of organ dysfunction. As the majority of mild cases recover with standard care, this demographic represents the most urgent unmet need and necessitates a meticulous evaluation of the potential risk-benefit ratio of the combination, utilizing levofloxacin as the primary candidate. This signifies the subsequent crucial stage in improving results for those suffering from these challenging infections, and we firmly endorse the initiation of such clinical trials.
